# Prognostic Value of Tumor-Stroma Ratio in Rectal Cancer: A Systematic Review and Meta-analysis

**DOI:** 10.3389/fonc.2021.685570

**Published:** 2021-05-26

**Authors:** Yuzhou Zhu, Zechuan Jin, Yuran Qian, Yu Shen, Ziqiang Wang

**Affiliations:** ^1^ Department of Gastrointestinal Surgery, West China Hospital, Sichuan University, Chengdu, China; ^2^ West China Hospital of Stomatology, Sichuan University, Chengdu, China

**Keywords:** tumor-stroma ratio, prognosis, rectal cancer, meta-analysis, systematic review

## Abstract

**Background:**

Tumor-stroma ratio (TSR) is a promising new prognostic predictor for patients with rectal cancer (RC). Although several studies focused on this pathologic feature, results from those studies were still inconsistent.

**Methods:**

This research aimed to estimate the prognostic values of TSR for RC. A search of PubMed, EMBASE, and Web of Science was carried out. A meta-analysis was performed on disease-free survival, cancer-specific survival, and overall survival in patients with RC.

**Results:**

The literature search generated 1,072 possible studies, of which a total of 15 studies, involving a total of 5,408 patients, were eventually included in the meta-analysis. Thirteen of the 15 articles set the cutoff for the ratio of stroma at 50%, dividing patients into low-stroma and high-stroma groups. Low TSR (rich-stroma) was significantly associated with poorer survival outcome. (DFS: HR 1.54, 95% CI 1.32–1.79; OS: HR 1.52 95% CI 1.34–1.73; CSS: HR 2.05 95% CI 1.52–2.77).

**Conclusion:**

Present data support TSR to be a risk predictor for poor prognosis in RC patients.

## Introduction

Rectal cancer (RC), marked by its high mortality and morbidity, is the third most common cancer in the world (1). The American Joint Commission on Cancer (AJCC) Classification System (TNM) is the most commonly used system to determine the degree of cancer progression in clinical decision-making (2, 3) Unfortunately, clinical outcomes vary for patients with RC at the same TNM stage (4). Besides, the prognosis of some stage IIB RC patients is worse than that of stage IIIA, which leads to under-treatment of stage II patients and over-treatment of stage III patients (5–8). The current TNM system focuses on the anatomic feature, but additional prognostic and/or predictive markers are required (5). Based on tumor cell features, additional biomarkers have been suggested, involving molecular mechanisms, tumor cell structure, genetic mutations, tumor immune response, as well as gene expression (9). The high cost of transcriptomic and genetic data is a disadvantage of these methods, whereas traditional pathological analysis using a microscope is simple, inexpensive, and effective (9). A biomarker-based on microscopic analysis is thus desirable.

The tumor-stroma ratio (TSR), also known as the tumor-stroma percentage, is measured on traditional hematoxylin and eosin (H&E)-stained paraffin sections at the invasive tumor front. Patients with a high stroma were correlated to a poorer prognosis (10). A complex mixture of non-neoplastic cells, involving endothelial cells, fibroblasts, and immune cells embedded in the extracellular protein matrix (ECM), forms the tumor stroma (10). Stromal cells supply growth factors, metabolites, and cytokines to the tumor and facilitate the blood vessels development. In this way, the tumor stroma in cancer cells leads to tumorigenesis and EMT induction (11).

Several studies indicated that tumor stroma overgrowth could predict poor survival outcomes. These results were, however, contradicted by some scholars (12–15). Considering the need for new prognostic factors to better determine therapeutic strategies, we performed a meta-analysis to analyze TSR prognostic value in RC patients.

## Methods

### Search Strategy

We performed this meta-analysis according to the Preferred Reporting Items for Systematic Reviews and Meta-Analyses (PRISMA) (16) PICOS criteria and searched the databases of PubMed, Embase, and Web of Science up to April 2021. The search strategy was presented in **Table 1**.

**Table 1:** Search Strategy.

Database	Search Strategy
Pubmed	(stroma* OR Glasgow tumor microenvironment score) AND (“prognosis”[mesh] OR progno* OR predic* OR survival OR mortality) AND (“Rectal Neoplasms”[Mesh] OR Rectal Neoplasm OR Rectal Tumors OR Rectal Tumor OR Rectal Carcinoma OR Rectal Carcinomas OR Rectal Cancer)
Embase	(stroma* OR Glasgow tumor microenvironment score) AND (“prognosis”[mesh] OR progno* OR predic* OR survival OR mortality) AND (“Rectal Neoplasms”[Mesh] OR Rectal Neoplasm OR Rectal Tumors OR Rectal Tumor OR Rectal Carcinoma OR Rectal Carcinomas OR Rectal Cancer)
Web of Science	(stroma* OR Glasgow tumor microenvironment score) AND (progno* OR predic* OR survival OR mortality) AND (Rectal Neoplasm OR Rectal Tumors OR Rectal Tumor OR Rectal Carcinoma OR Rectal Carcinomas OR Rectal Cancer)

### Study Selection

The criteria for inclusion included: 1) the studies revealed the correlation between TSR and survival outcomes of RC patients, such as DFS, CSS, and OS; 2) the RC patients were only classified into two groups, namely, stroma-poor (high TSR) and stroma-rich (low TSR); 3) the HRs for survival outcomes were reported in the study directly or could be extracted from original data; 4) the studies were published in English or Chinese as full papers.

The exclusion criteria included: 1) studies researching the mechanism or functions; 2) studies whose available data was inaccessible; 3) reviews, conference abstracts, editorials, or letters;

If several studies used the same patient population, we chose the study with the largest sample size.

All included studies followed PICOS criteria. P: RC patients with TSR status; I & C: high TSR and low TSR; O: DFS, CSS and OS; S: retrospective or prospective studies on prognostic value of different status TSR.

### Data Extraction and Quality Assessment

According to the research selection criteria, two authors (YZ and ZJ) checked and extracted information from all included studies independently. Any dispute was settled by consensus among the reviewers. The authors’ first name, research area, sample size, publication year, cutoff value, clinical characteristics, survival results, HR estimate, and quality scores were extracted from the studies. In articles where both univariate and multivariate analyses for the HRs and 95% Cis were conducted, we only applied the latter to the data synthesis, since it was more reliable and took the confounding factors into account (17). The one with the largest sample size or the smallest heterogeneity was applied to data synthesis in studies where different HRs were identified by various TSR detection methods (18). HR was derived from a univariate analysis or calculated by the Kaplan-Meier survival curve in the absence of data from multivariate analysis (19).

NOS scoring system was adopted to measure the quality of included studies (20). The total NOS score varied from 0 to 9, and studies were considered as high quality if at least six scores were reached.

### Statistical Analysis

Based on data from included studies, the predictive value of TSR to survival endpoints (DFS, CSS, and OS) was measure by the combined HR and 95% CI. HR > 1 with 95%CI exceeding 1 demonstrated an increased risk of poor prognosis for patients with stroma-rich RC (21). Z-test was performed to assess the statistical significance of pooled HR. Statistically significant results were considered if P < 0.05 (21). The odds ratios (ORs) and their corresponding 95% CIs were pooled to evaluate the correlation between TSR and clinicopathological characteristics (22). (i.e., histological grade, lymph node metastasis, depth of invasion, and lymphatic or vascular invasion). All statistical analyses were conducted by STATA version 16.0 (STATA Corporation, College Station, TX, USA). The statistical analyses were all two-sided. The presumption of heterogeneity was tested based on the Q statistics *via* the chi-squared test and was considered statistically significant at P<0.05 (23).

In this meta-analysis, a random-effects model (DerSimonian and Laird method) was adopted to calculate the pooled HR, if significant poor heterogeneity was observed among the articles (P<0.05 or I2 > 50%) (24). Otherwise, a model of fixed effects was used (25). The sensitivity analysis was performed by omitting each study to verify the stoutness of the pooled HRs. Publication bias was evaluated by Begg’s and Egger’s asymmetry tests (26). A two-tailed P value of less than 0.05 was identified as statistically significant.

## Results

### Description of Included Studies

**Figure 1:**
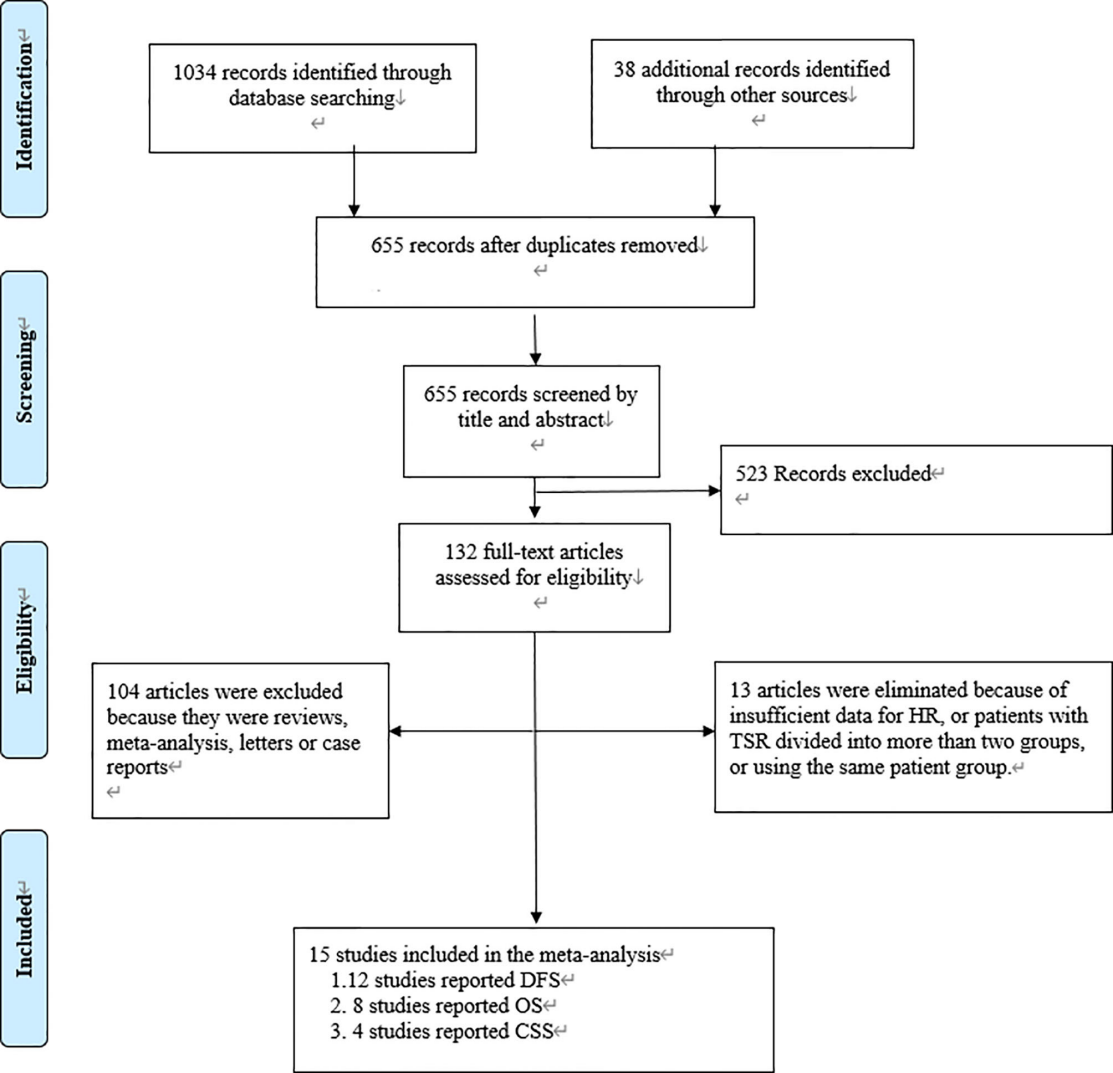
Flowchart according to PRISMA.

We found 1,072 articles at initial searching and omitted 417 articles as duplicates. After reviewing the titles and abstracts, we excluded another 523 articles, leaving 132 articles for further assessment. Consequently,15 studies involving 5048 patients met the criteria for inclusion and were included in the meta-analysis (12–15, 27–37) (**Figure 1** **)**. The number of patients in each study ranged from 65 to 1212. Among these 15 studies, 12 studies reported DFS as survival outcome (12–15, 27–32, 35, 37), four reported CSS (14, 33, 34, 36) and eight reported OS (13, 15, 27, 28, 31, 32, 35, 37). Thirteen of the 15 articles had set the cutoff at 50% to divide patients into low-stroma and high-stroma groups (12, 14, 15, 27–35, 37), while one at 47% (36) and the other at 68% (13). The NOS scores of all studies included were > 6 in terms of methodological consistency. Detailed features of all included studies were shown in **Table 2**.

**Table 2:** Characteristics of 15 studies included in the meta-analysis.

Study	Country	Clinical stage	Duration	No. of patients (M/F)	Cut off	Stroma low/rich	Outcome,* HR (95% CI)	Analysis	Outcome,* HR (95% CI)	Analysis	Quality
Dang et al, (12)	The Netherlands	I	2000–2014	223 (125/98)	50%	156/63	DFS	MV	NR	NR	8
							0.66 (0.37–1.18)				
Yang et al, (28)	China	II	2009–2015	188 (121/67)	50%	153/35	DFS	UV	OS	UV	8
							1.78 (0.86–3.71)		1.51 (0.64–3.57)		
Zengin and Benek, (27)	Turkey	III-IV	2004–2014	172 (89/83)	50%	94/78	DFS	MV	OS	MV	8
							1.32 (1.17–2.55)		1.37 (1.25–2.56)		
Geessink et al, (14)	Sweden	I-III	1996–2006	119 (33/86)	50%	87/42	DFS	MV	CSS	MV	7
							1.42 (0.77–2.61)		1.76 (0.93–3.34)		
Zengin, (13)	Turkey	I	1998–2005	88 (53/35)	68%	36/53	DFS	MV	OS	MV	8
							1.50 (1.11–1.91)		1.42 (1.10–1.82)		
Eriksen et al, (31)	Denmark	II	NR	573 (284–289)	50%	404/169	DFS	MV	OS	MV	7
							1.34 (1.01–1.79)		1.38 (1.02–1.86)		
Zunder et al, (15)	The Netherlands	II-III	2004–2007	1212(673/539)	50%	824/339	DFS	UV	OS	UV	8
							1.75 (1.32–2.33)		1.54 (1.04–2.29)		
Huijbers et al, (35)	The Netherlands	II-III	2005–2010	965 (548/417)	50%	642/323	DFS	MV	NR	NR	7
							1.52 (1.18–1.96)				
Flam et al, (32)	Croatia	I-IV	2006–2007	236 (284/289)	50%	131/105	DFS	UV	OS	UV	8
							1.37 (0.94–1.99)		1.26 (0.77–2.05)		
Hansen et al, (30)	Denmark	II-III	2010–2013	65 (35/30)	50%	33/29	DFS	UV	NR	NR	7
							5.38 (1.08–26.83)				
Park et al, (33)	The UK	I-III	1997–2008	246 (129/117)	50%	179/67	NR	NR	CSS	MV	8
									2.36 (1.44–3.84)		
Huijbers et al, (35)	The Netherland	II-III	2002–2004	710 (438/272)	50%	503/207	DFS	MV	OS	MV	7
							1.95 (1.45–2.61)		1.71 (1.22–2.41)		
Park et al, (34)	The UK	I-III	1997–2008	331 (160/171)	50%	250/81	NR	NR	CSS	MV	6
									1.84 (1.17–4.54)		
West et al, (36)	The UK	I-IV	1990–1995	145 (58/87)	47%	110/35	NR	NR	CSS	MV	7
									2.09 (1.09–4.00)		
Mersker et al, (37)	The Netherlands	I-II	1980–2001	135 (74/61)	50%	101/34	DFS	MV	OS	MV	6
							2.43 (1.55–3.82)		2.73 (1.73–4.30)		

No. of patients, number of patients; M, male; F, female; HR, hazard ratio; CI, confidence interval; DFS, disease-free survival; OS, overall survival; UV, univariate analysis; MV, multivariate analysis; NR, not reported

*Survival outcomes of the stroma-poor group served as the control group (HR = 1).

### Prognostic Value of TSR on DFS

**Figure 2:**
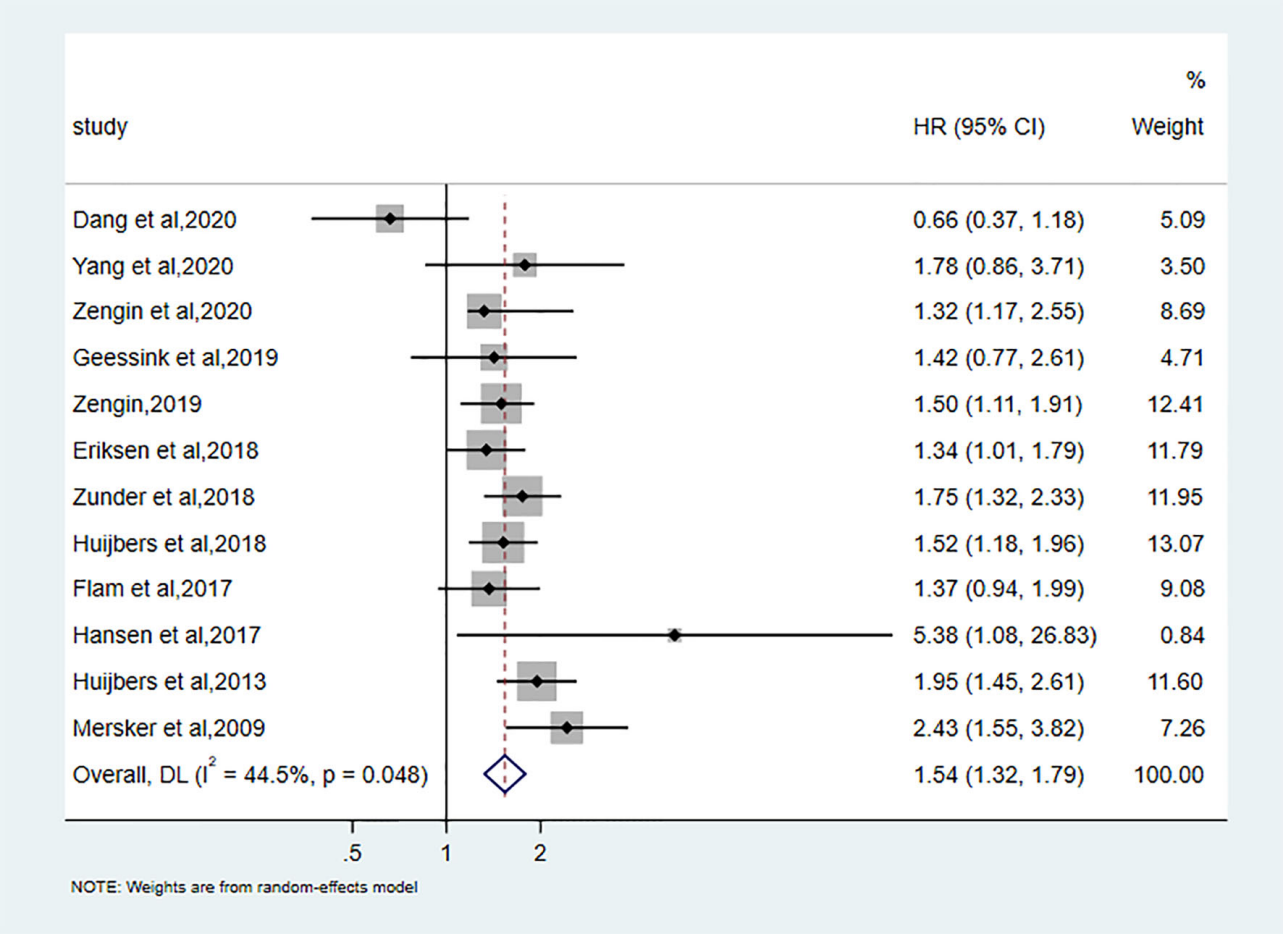
Forest plots for DFS.

Twelve studies discussed the correlation between TSR and DFS. A total of 4645 patients to assess this relationship revealed that low TSR (stroma-rich) was significantly associated with the bad outcome of DFS (pooled hazard ratio [HR]:1.54, 95%CI: 1.32–1.79, P < 0.001; random effects, I2 = 44.5%, Ph = 0.048) (**Figure 2**).

### Prognostic Value of TSR on OS and CSS

**Figure 3:**
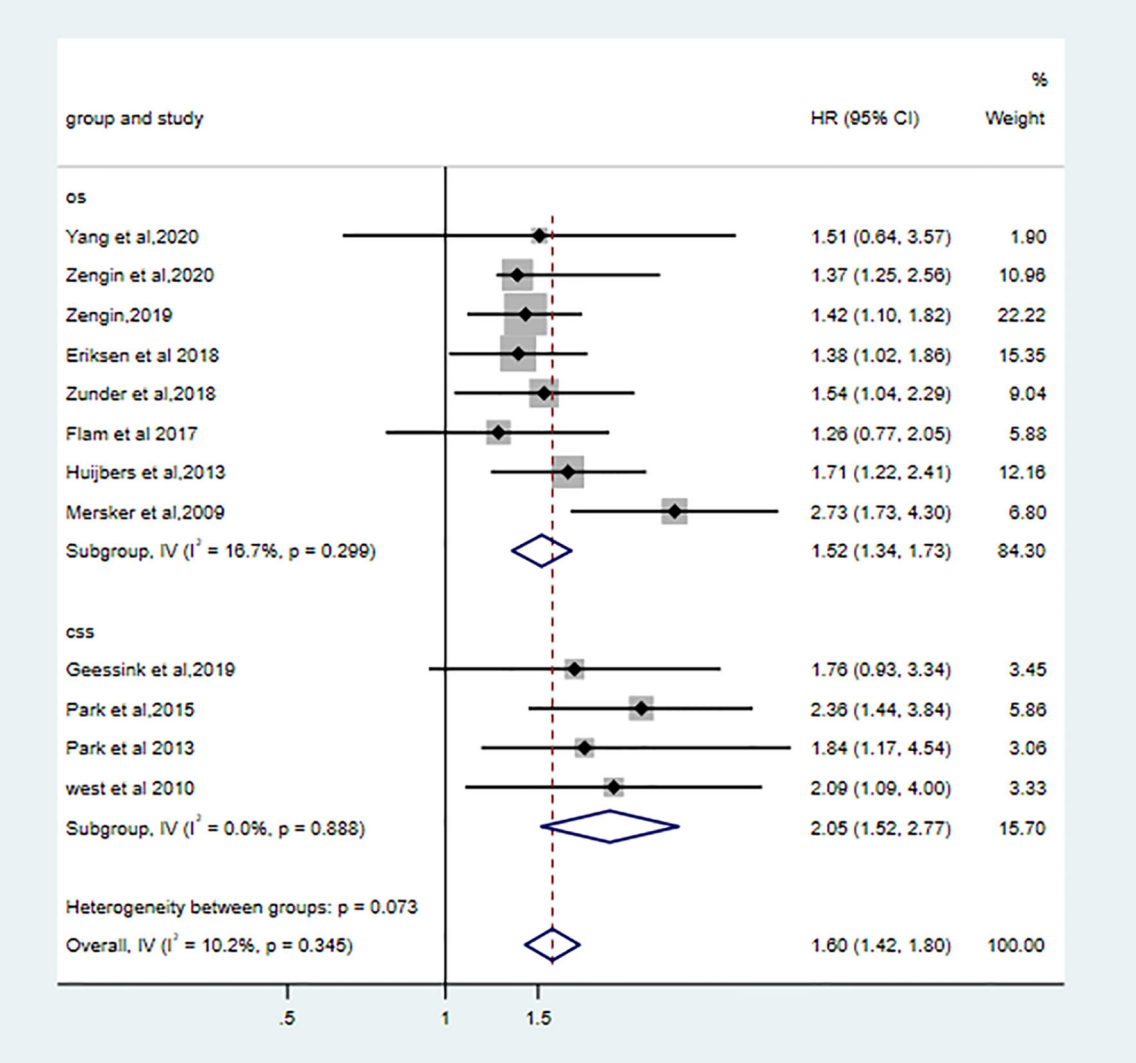
Forest plots for CSS and OS.

Eight qualified studies correlated low TSR with poor OS. (pooled HR: 1.52, 95% CI: 1.34–1.73, P < 0.001, I2 = 16.7%, Ph = 0.30). Four studies showed high stroma was associated worse CSS (pooled HR: 2.05, 95%CI: 1.52–2.77, I2 = 0.0%, Ph = 0.89). High stroma was still correlated with poor OS (the combined HR: 1.60 95%CI:1.42–1.80, fixed effects, I2 = 10.2%, p=0.35) (**Figure 3**).

### Correlation Between Clinicopathological Characteristics and TSR

In order to explore the relationship between clinicopathological characteristics and TSR, we tested some basic factors (**Table 3**). Our results showed that a low TSR was significantly associated with venous invasion (negative vs positive OR: 0.72, 95%CI: 0.57–0.92, P = 0.009, fix effects, I2 = 0%, Ph = 0.71). Other variables, including differentiation (moderate + well/or poor), or lymph node status (pos/neg), or tumor invasion (T1 + T2/T3 + T4) were not observed to be correlated with TSR.

**Table 3:** Meta-analysis of TSR and clinical-pathological characteristics in RC patients.

Characteristics	No. of studies	No. of patients	OR (95%CI)	I2(%)	Ph	Z	P
Histological grade (moderate/well vs. poor)	5	1223	1.26 (0.75–2.11)	44	0.13	0.86	0.39
Venous invasion (negative vs. positive)	3	1401	0.72 (0.57–0.92)	0	0.71	2.61	0.009
Lymph node status (negative vs. positive)	6	1831	0.65 (0.41–1.04)	64	0.02	1.79	0.07
Tumor invasion (T1–2 vs. T3–4)	5	1087	1.00 (0.53–1.88)	71	0.008	0.00	1.00

OR, odds ratio; Ph, p value for heterogeneity based on Q test; P, p value for statistical significance based on Z test.

### Sensitivity Analysis

**Figure 4:**
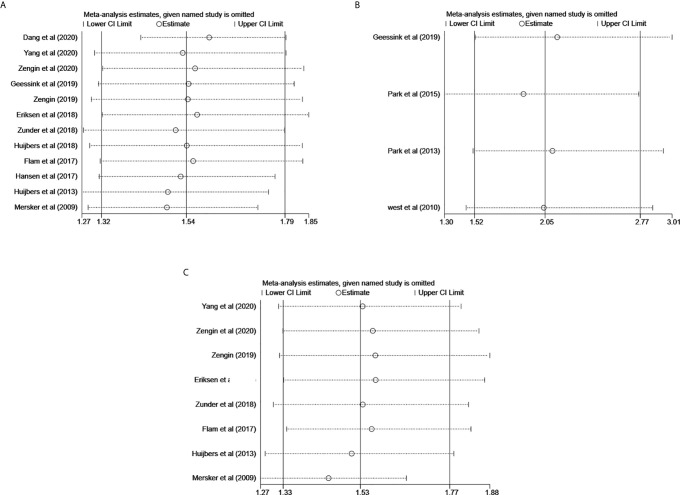
Effect of individual studies on pooled HR for the prognostic value of TSR on RC. **(A)** Sensitivity analysis for DFS. **(B)** Sensitivity for CSS. **(C)** Sensitivity analysis for OS.

No point estimate of the omitted individual dataset exceeded the 95% CI of the combined overall HR of DFS (**Figure 4A**), CSS (**Figure 4B**), and OS (**Figure 4C**), which suggested that the meta-analysis results were not dominated by any individual study, thus the results were consistent and accurate.

### Publication Bias

We did not find publication bias for DFS. (Begg’s test, P = 0.837; Egger’ test, P = 0.681). Hence, the results were consistent and reliable. As for CSS or OS, the publication bias was not conducted because <10 qualified studies were reported.

## Discussion

In this study, we performed a meta-analysis to evaluate the prognostic value of TSR in RC patients, since the clinical value of TSR remained unclear. According to the results, a rich-stroma was significantly correlated to unfavorable prognosis (DFS, CSS, and OS) in RC. Therefore, in patients with RC, TSR could be an effective predictor of DFS, CSS, and OS. Furthermore, we evaluated the correlation between TSR and other clinicopathological characteristics which have been proven of prognostic value for RC patients. According to our pooled results, the abnormal proportion of TSR was significantly related to several clinical factors, such as venous invasion, indicating that tumor-related stroma played an important part in promoting tumor progression. Although several previous studies focused on this area, the results of those studies were still inconsistent (12–15). The reasons could be the possible subjectivity of TSR evaluation, and patients included in different studies were different This was the first meta-analysis.

Before conclusions on the outcomes, certain strengths and weaknesses should be considered. The advantages of this review included extensive literature retrieval and strict inclusion criteria, which were helpful to include all potentially eligible studies. Moreover, although moderate heterogeneity (I2 = 16.7%) was observed in DFS analysis, heterogeneity in CSS analysis (I2 = 0.0%), and OS analysis (I2 = 16.7%) were slight.

Most of the studies were of high quality, two studies by the same author contained the same patient group (37, 38), thus the one with the larger sample size was included (37). Finally, four out of 12 studies did not report DFS analysis adjusted for confounding factors (15, 28, 30, 32) [three out of eight OS studies (15, 28, 32)], which indicated that there were some residual confounding factors in combined HR. Two studies did not report HRs for DFS directly (30, 32) (while two for OS (30, 32)), we estimated HR from KM curve by the methods by Parmar et al (19). This could explain the unprecise of their 95% CI. Geessink et al. estimated stroma ratio by both visual and auto-methods (14), we included the results from the visual method to reduce heterogeneity among the included literature. West et al. and Zengin et al. defined rich-stroma as stroma more than 47% and 68%, respectively (27, 36), while the rest included studies all set the cutoff point at 50%. Despite the above problems, Sensitivity analysis showed that no individual study had statistically significant influence on the pooled results.

The knowledge of the stroma of tumors has increased in recent years (39–41). Cancer-associated fibroblasts (CAFs) in tumor stroma tend to be able to promote tumor growth by tuning the normal stromal microenvironment from being tumor suppressive to tumor supportive (41). However, the mechanism of stroma in the progression of RC is not completely clear. Tumor cells infiltrate the basement membrane and stimulate stromal cells to establish a tumor microenvironment at the early stage of tumor invasion (40). Although stromal cells are not malignant, they interact with surrounding cancer cells or other stroma cells, resulting in irregular phenotypic and functional changes (39). Furthermore, these modifications cause immune and endothelial cell recruitment, proteolysis, matrix remodeling, cell adhesion loss, and cytoskeletal rearrangements, which are all important processes in tumor progression (42). Besides, the tumor-activated stroma facilitate the immune evasion of malignant cells, the disturbance of epithelial tissue, and tumor invasion (43)

Tumor-related stroma components, including extracellular matrix (ECM), various secreted factors, and multiple cell types, are diverse. As an intermediate, the ECM allows cancer cells to communicate with stromal cells to colonize the microenvironment and metastasize (44). Although stroma therapies have not yet been clinically implemented, they may become critical in future. If such therapies were available, it would be necessary to decide if these therapies would favor patients with low TSR over those with high TSR. According to this meta-analysis, TSR is an important predictor in RC. It could be used to assess patient prognosis after surgery and should be taken into consideration for postoperative treatment planning.

Although this meta-analysis showed promising findings, it had some limitations. First, this study found a low heterogeneity, but it could have biases, one of which was the TSR assessment process. Although those studies had standard TSR assessment methods, personal subjectivity could still not be avoided. Therefore, a more systematic and scientific method is required to test TSR. Secondly, this study only included original reports published in English and Chinese.

## Conclusion

In conclusion, our meta-analysis indicates that rich stroma is a poor prognosis predictor for DFS CSS and OS, in RC patients. TSR can be conveniently used as a prognostic marker to help in the decision making for adjuvant therapy.

## Data Availability Statement

The original contributions presented in the study are included in the article/supplementary material. Further inquiries can be directed to the corresponding author.

## Author Contributions

YZ and ZJ contributed equally to this article. YZ designed the project, developed the search strategy, and wrote the manuscript. ZJ checked the search and reviewed the manuscript. YQ performed literature screening and data extraction and conducted the quality assessment of the included studies. YS carried out the data analysis. ZW reviewed the manuscript and finally approved the version to be published. All authors contributed to the article and approved the submitted version.

## Funding

The work is supported by the Department of Science and Technology of Sichuan Province No. 2018RZ0091.

## Conflict of Interest

The authors declare that the research was conducted in the absence of any commercial or financial relationships that could be construed as a potential conflict of interest.

## Abbreviations

TSR, tumor-stroma ratio; RC, rectal cancer; AJCC, American Joint Commission on Cancer.
